# Deep learning based automatic detection algorithm for acute intracranial haemorrhage: a pivotal randomized clinical trial

**DOI:** 10.1038/s41746-023-00798-8

**Published:** 2023-04-07

**Authors:** Tae Jin Yun, Jin Wook Choi, Miran Han, Woo Sang Jung, Seung Hong Choi, Roh-Eul Yoo, In Pyeong Hwang

**Affiliations:** 1 Institute of Radiation Medicine, Seoul National University Medical Research Center, Seoul, Republic of Korea; 2 Department of Radiology, Seoul National University Hospital, Seoul, Republic of Korea; 3 Department of Radiology, Ajou University School of Medicine, Suwon, Republic of Korea

**Keywords:** Clinical trials, Brain imaging, Computed tomography

## Abstract

Acute intracranial haemorrhage (AIH) is a potentially life-threatening emergency that requires prompt and accurate assessment and management. This study aims to develop and validate an artificial intelligence (AI) algorithm for diagnosing AIH using brain-computed tomography (CT) images. A retrospective, multi-reader, pivotal, crossover, randomised study was performed to validate the performance of an AI algorithm was trained using 104,666 slices from 3010 patients. Brain CT images (12,663 slices from 296 patients) were evaluated by nine reviewers belonging to one of the three subgroups (non-radiologist physicians, *n* = 3; board-certified radiologists, *n* = 3; and neuroradiologists, *n* = 3) with and without the aid of our AI algorithm. Sensitivity, specificity, and accuracy were compared between AI-unassisted and AI-assisted interpretations using the chi-square test. Brain CT interpretation with AI assistance results in significantly higher diagnostic accuracy than that without AI assistance (0.9703 vs. 0.9471, *p* < 0.0001, patient-wise). Among the three subgroups of reviewers, non-radiologist physicians demonstrate the greatest improvement in diagnostic accuracy for brain CT interpretation with AI assistance compared to that without AI assistance. For board-certified radiologists, the diagnostic accuracy for brain CT interpretation is significantly higher with AI assistance than without AI assistance. For neuroradiologists, although brain CT interpretation with AI assistance results in a trend for higher diagnostic accuracy compared to that without AI assistance, the difference does not reach statistical significance. For the detection of AIH, brain CT interpretation with AI assistance results in better diagnostic performance than that without AI assistance, with the most significant improvement observed for non-radiologist physicians.

## Introduction

Acute intracranial haemorrhage (AIH) is a life-threatening disease with a 30-day mortality rate ranging from 35% to 52%. Most notably, only 20% of survivors are expected to achieve full functional recovery at 6 months^1–3^. Magnetic resonance imaging (MRI) scans may be as accurate as CT scans with regard to the detection of AIH in patients presenting with acute focal stroke symptoms^4^ and are more accurate than CT scans in terms of detecting microhaemorrhage. Nevertheless, non-contrast brain CT scans are the most widely used first-line diagnostic approach for identifying AIH due to the several disadvantages of MRI scans, including their limited availability, long image acquisition times, high cost, and issues with patient tolerance^5,6^

Despite the clinical relevance of diagnosing AIH using brain CT scans—false negatives may delay correct diagnosis, which can cause devastating consequences, whereas false positives will lead to unnecessary examinations—prompt and accurate assessment of AIH using brain CT scans remains a challenge for physicians. In addition, the high volumes of imaging data that require assessment place a significant burden on radiologists who need to maintain diagnostic accuracy and efficiency^7,8^.

Over the past decade, deep learning-based artificial intelligence (AI) technology has made significant advances with improvements in computer power and accumulation of ‘big data’. Advances in deep learning-based image recognition, as a part of machine learning, are transforming the medical field and have the potential to further improve the processes in the medical imaging domain^9^. These innovations may increase diagnostic accuracy, enable prompt diagnosis and improved management of various conditions, and facilitate new biological insights. Various AI algorithms for AIH diagnosis have been developed and shown promising results in the detection, classification, quantification, and prediction of AIH using brain CT scans^7,8,10–15^.

Previous studies employing deep learning architectures have predominantly used haemorrhage detection methods based on labelling or segmentation by experts^7,8,10,11,13,15–17^. However, the classification of AIH is contingent on the opinion of experts, and the training of the system depends on the labelling of AIH-suspected areas by experts. As such, discordance between experts regarding the final classifications or labelling of images is inevitable. In addition, poorly defined characteristics, variability in sizes and morphologies, and the attenuation of AIH contribute to inter-observer discordance even between expert neuroradiologists. In this regard, an anomaly detection process based on unsupervised training alongside a haemorrhage detection process can overcome the drawbacks of the supervised haemorrhage detection process used in conventional AI algorithms for intracranial haemorrhage detection, leading to an improvement in diagnostic performance^18–22^. In terms of deep learning architectures used for haemorrhage detection, the majority of previous investigations have relied on convolutional neural network (CNN)-based AI algorithms that have been reported to classify and quantify intracranial haemorrhages with good diagnostic performance^11,13,23–26^. Recent studies have proposed new deep learning architectures based on a joint recurrent neural network (CNN-RNN) approach with promising results, highlighting its potential for assisting radiologists and physicians in their clinical diagnosis workflow^15,27^.

Although the excellent performance of deep learning-based AI algorithms has been proven in the internal validation cohort, achieving persistent favourable results without performance decline in the external validation dataset consisting of a diverse patient population and scanner remains challenging^28,29^.

In this study, we developed a deep learning-based automatic detection AI algorithm for identifying AIH on brain CT scans based on a new approach that combined haemorrhage detection (based on a joint CNN-RNN system) and anomaly detection (based on unsupervised training) using a large dataset. We evaluate the diagnostic performance of this AI algorithm in a large external validation dataset to validate our approach and also conduct a retrospective multi-reader study to validate the improvement in the diagnostic performance with the assistance of our AI algorithm by clinicians of varying expertise levels.

## Results

### Diagnostic performance of the AI-based diagnostic support software in the external validation dataset

The overall AUROC for AI performance in the external validation dataset was 0.992 and 0.977 for patient-wise and slice-wise analyses, respectively. The patient and slice-wise analyses indicated a sensitivity of 94.4% and 79.0% and a specificity of 98.2% and 99.3%, respectively. Details regarding the results for external validation are presented in Table 1 and Supplementary Tables 1–3.

**Table 1:** Diagnostic performance of AI in the external validation set (full analysis set: 49,841 patients, 1,855,465 slices).

	Accuracy	Recall (sensitivity)	Precision (PPV)	F1 score	Specificity	AUC	NPV
Patient-wise(*N* = 49,841)	0.977	0.944	0.894	0.913	0.982	0.992	0.992
Slice-wise (*N* = 1,855,465)	0.985	0.790	0.832	0.810	0.993	0.977	0.991

*PPV* positive predictive value, *AUC* area under the receiver-operating curve, *NPV* negative predictive value.

### Evaluation of the diagnostic performance of the AI-based diagnostic support software

**Fig. 1:**
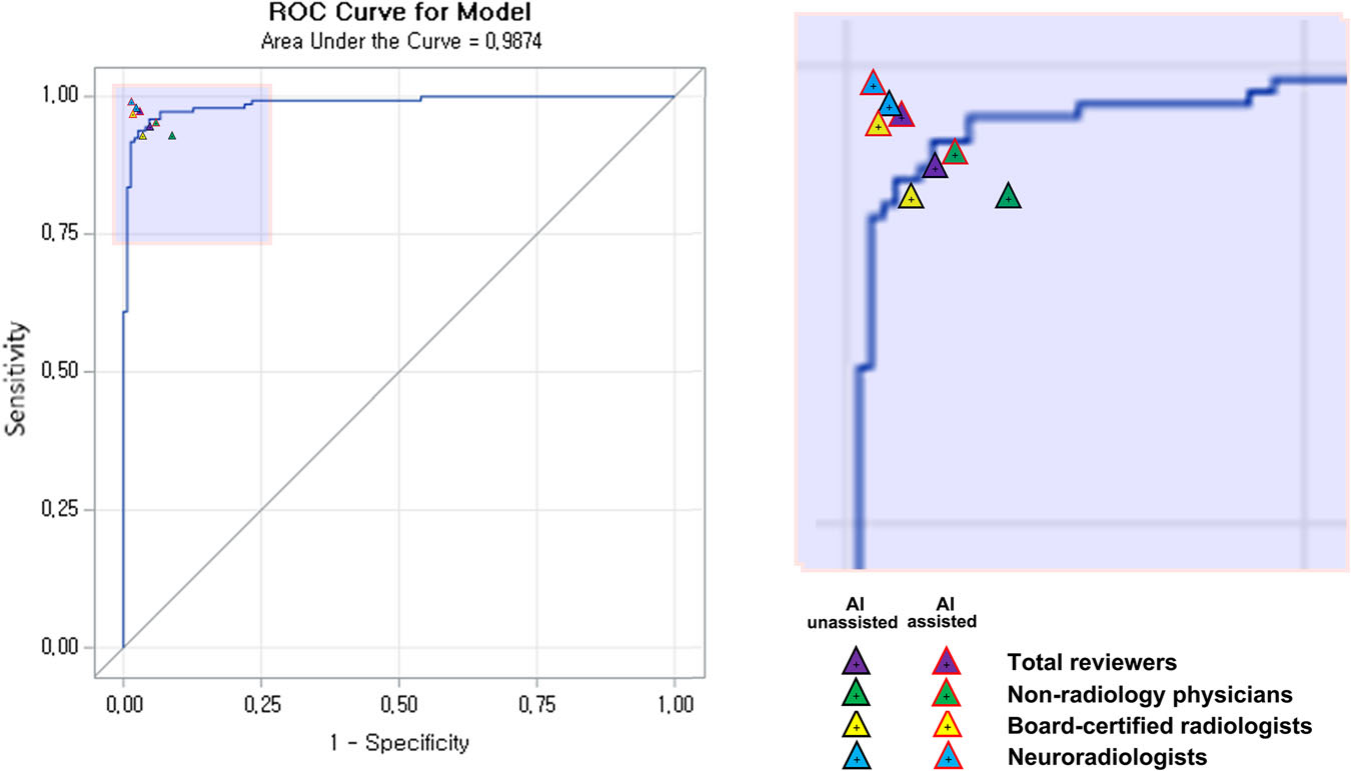
Diagnostic performance of reviewers in terms of basic ROC curves for patient-wise AI standalone performance. In the reader assessment study, the AI-assisted group demonstrated significantly higher diagnostic accuracy in AIH detection compared to the AI-unassisted group in the patient-wise analysis (0.9703 [95% CI: 0.9632, 0.9765] vs. 0.9471 [95% CI: 0.9379, 0.9553], *p* < 0.0001). Based on subgroup analysis, non-radiologist physicians achieved the greatest benefit in terms of improvement in diagnostic accuracy with AI assistance relative to that for the AI-unassisted group (0.9505 [95% CI: 0.9340, 0.9638] vs. 0.9189 [95% CI: 0.8990, 0.9360], with an improvement of 3.15 [95% CI: 0.86, 5.45], *p* = 0.0072) for non-radiologist physicians to the level of radiologists without AI assistance (0.9459 [95% CI: 0.9290, 0.9599]). In addition, board-certified radiologists demonstrated a significant improvement in diagnostic accuracy with AI assistance relative to that for the AI-unassisted group (0.9741 [95% CI: 0.9614, 0.9835] vs. 0.9459 [95% CI: 0.9290, 0.9599], with an improvement of 2.82 [95% CI: 1.00, 4.63], *p* = 0.0025), with an improvement for board-certified radiologists to the level of neuroradiologists without AI assistance (0.9764 [95% CI: 0.9641, 0.9853]). Note. ROC: receiver operating characteristic.

**Fig. 2:**
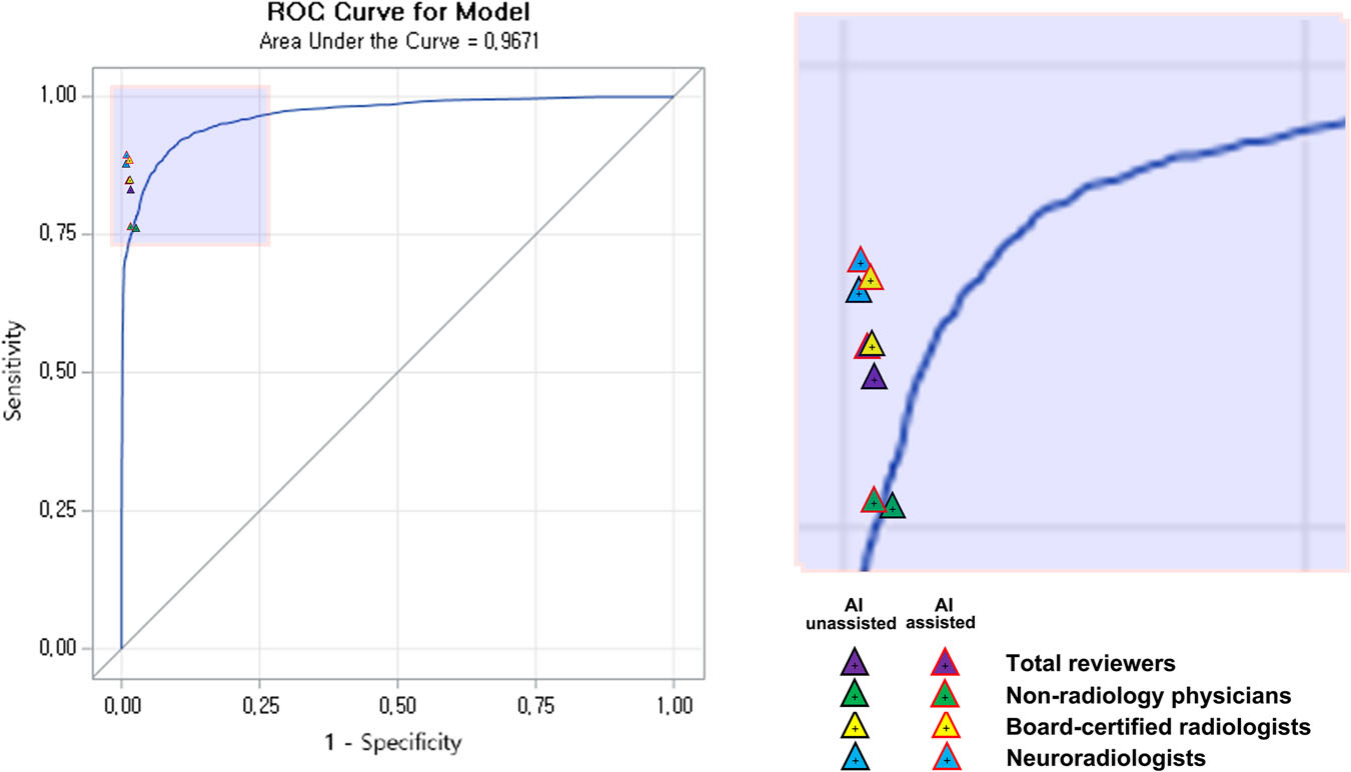
Diagnostic performance of reviewers in terms of basic ROC curves for slice-wise AI standalone performance. In the reader assessment study, the AI-assisted group demonstrated a significantly higher diagnostic accuracy in detecting AIH than that of the AI-unassisted group in the slice-wise analysis (0.9581 [95% CI: 0.9569, 0.9592] vs. 0.9522 [95% CI: 0.9509, 0.9534], *p* < 0.0001). Based on the subgroup analysis, non-radiologist physicians and board-certified radiologists demonstrated a significant improvement in diagnostic accuracy with AI assistance relative to that for the AI-unassisted group (for non-radiologist physicians: 0.9393 [95% CI: 0.9369, 0.9417] vs. 0.9306 [95% CI: 0.9280, 0.9332], with a difference of 0.87 [95% CI: 0.52, 1.22], *p* < 0.0001, for board-certified radiologists 0.9632 [95% CI: 0.9623, 0.9661] vs. 0.9567 [95% CI: 0.9546, 0.9587], with a difference of 0.75 [95% CI: 0.48, 1.03], *p* < 0.0001). Note. ROC: receiver operating characteristic.

**Fig. 3:**
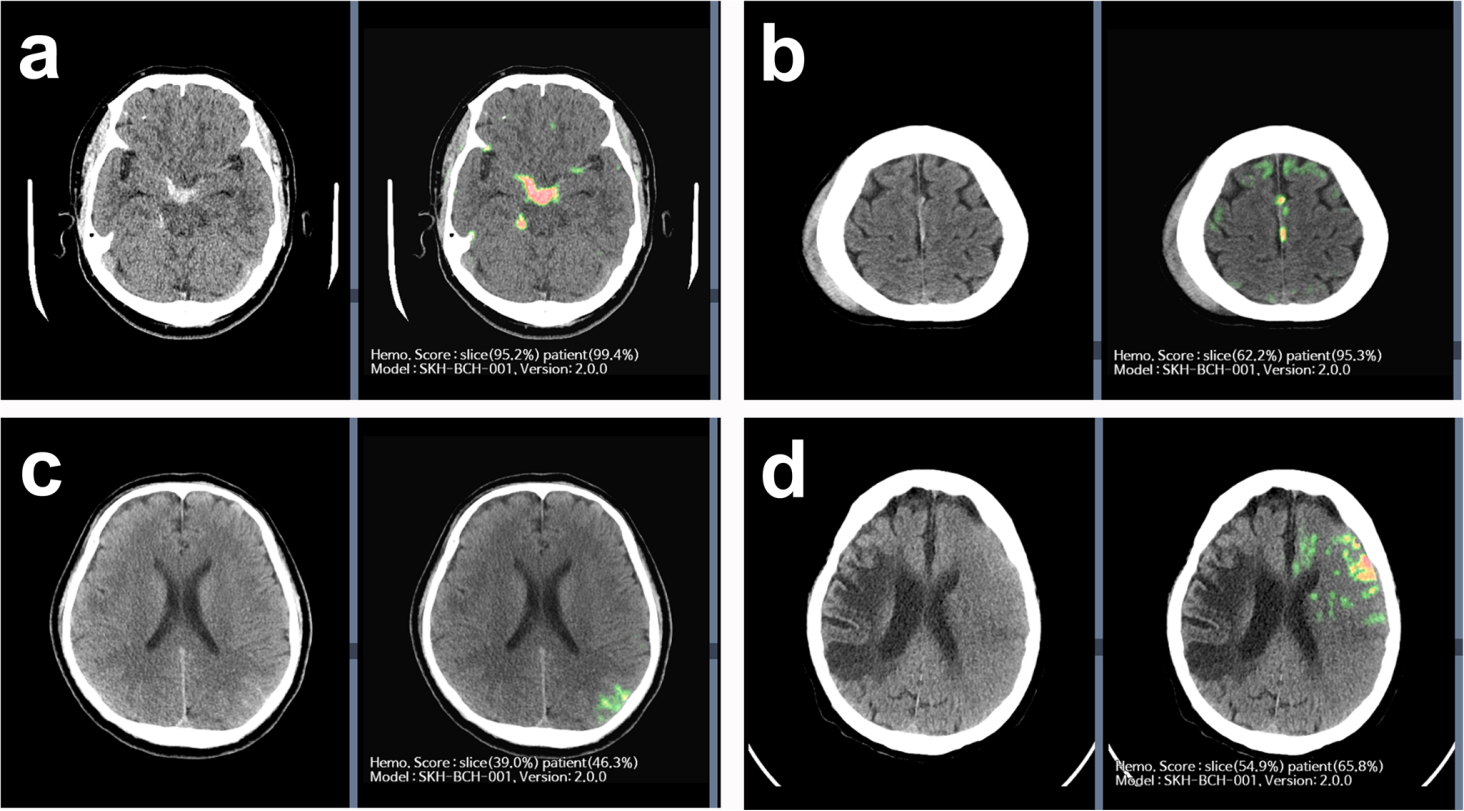
Representative images of AIH detection. **a** AI-assisted brain CT revealed probable AIH location as the basal cistern and right ambient cistern. AI-assisted brain CT provided AIH probability scores in a slice-wise (95.8%) and patient-wise (99.4%) manner. All nine reviewers agreed with the AIH diagnosis for both AI-unassisted and AI-assisted interpretations. **b** AI-assisted brain CT revealed the probable AIH location as the left side of the falx. AI-assisted brain CT provided the AIH probability scores in a slice-wise (62.2%) and patient-wise (95.3%) manner. For interpretation without AI assistance, one reviewer (non-radiologist physician) missed this case of AIH on the left side of the falx. All nine reviewers agreed with the AIH diagnosis for both AI-unassisted and AI-assisted interpretations. **c** AI-assisted brain CT revealed probable AIH location as the left parietal sulci. AI-assisted brain CT provided AIH probability scores in a slice-wise (39.0%) and patient-wise (46.3%) manner. For interpretation without AI assistance, two-thirds of the reviewers (three non-radiologist physicians, two board-certified radiologists, and one neuroradiologist) missed this case of AIH in the left parietal sulci. With the use of AI assistance, these six reviewers were able to correctly revise their decisions. **d** AI-assisted brain CT revealed the probable AIH location as the left frontal area. AI-assisted brain CT provided the AIH probability scores in a slice-wise (54.9%) and patient-wise (65.8%) manner. For the interpretation without AI assistance, one-third of the reviewers (one non-radiologist physician and two board-certified radiologists) reported it as an AIH. With the use of AI assistance, an additional one-third of the reviewers (one non-radiologist physicians, one board-certified radiologist, and one neuroradiologist) reported this as an AIH. However, the subtle hyperattentuating lesion in the left frontal area was due to the beam-hardening artefact of the skull.

**Fig. 4:**
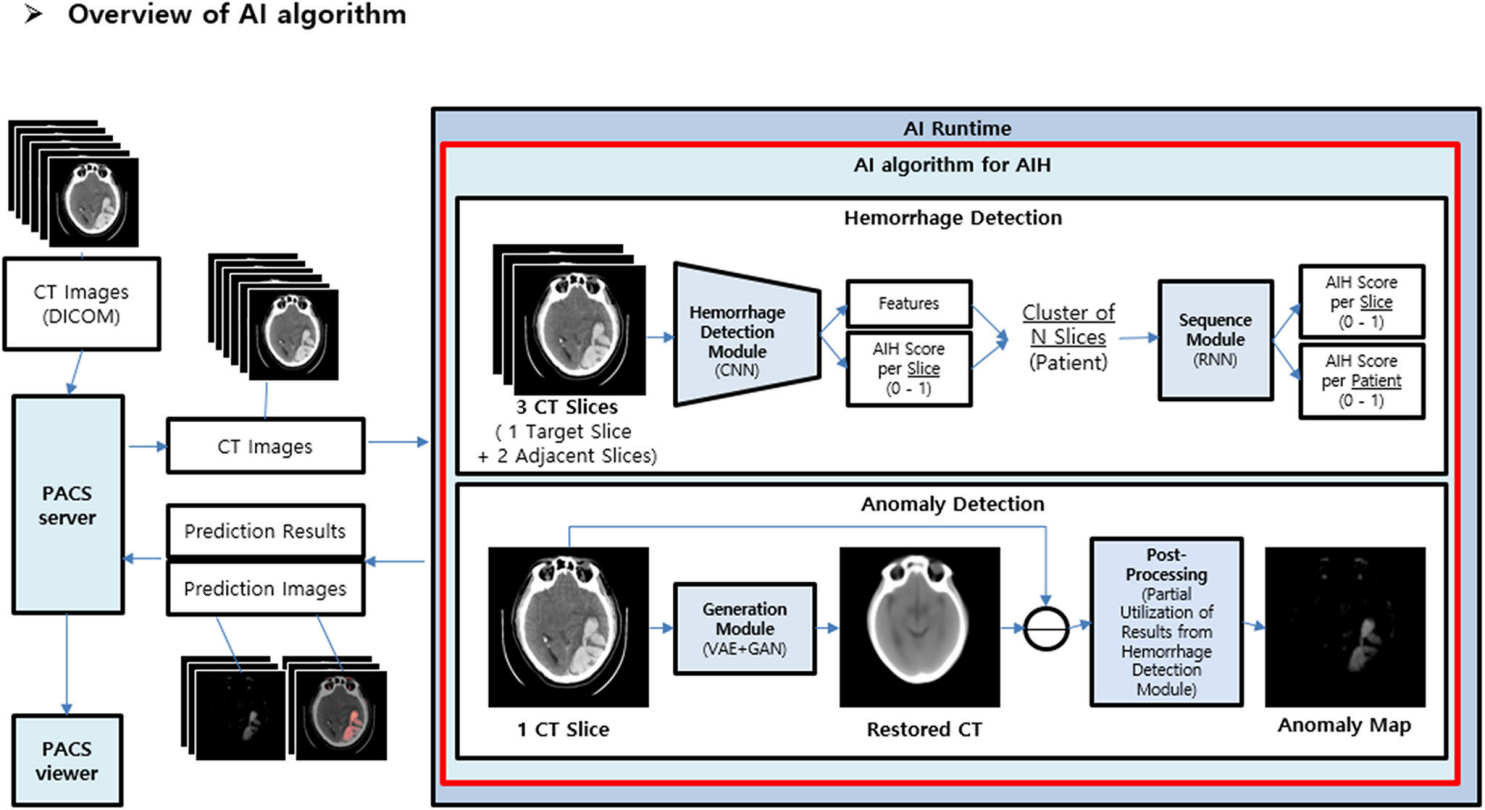
Overview of the AI algorithm. The diagram shows the architecture of the proposed AI algorithm. This new AI algorithm combined a supervised haemorrhage detection process and an unsupervised anomaly detection process. In addition, a combined CNN-RNN architecture was applied in the haemorrhage detection process. The presence or absence is determined through the haemorrhage detection process. As a result of this haemorrhage detection process, the AI algorithm provides the AIH score in the patient-wise and slice-wise manner. The AI algorithm provides the anomaly map for AIH patients through the subtraction between the original CT image and restored CT image (artificially generated normal image based on the unsupervised training from normal dataset) and postprocessing. The average additional time to access the AI-assisted CT images on PACS viewer was 97.4 seconds. Time from PACS server to AI, AI processing time, and time from AI to PACS viewer were 54.6 seconds (range, 37–91 seconds), 11.8 seconds (range, 0.8–90.6 seconds), 31.0 seconds (range, 30–33 seconds). Note. AIH acute intracranial haemorrhage, PACS picture archiving and communication system, CNN convolutional neural network, RNN recurrent neural network, VAE variational autoencoder, GAN generative adversarial network.

The overall AUROC for AI standalone performance in the dataset for the reader assessment study was 0.9874 and 0.9671 for patient-wise and slice-wise analyses, respectively (Figs. 3 and 4). For patient-wise analysis, the best diagnostic performance was achieved with a cut-off level of 39.84%, sensitivity of 95.89%, and specificity of 95.33%. For slice-wise analysis, the best diagnostic performance was achieved with a cut-off level of 7.70%, sensitivity of 89.87%, and specificity of 91.60%. At a cut-off level of 50.0%, the sensitivity and specificity were 93.84% and 97.33%, respectively, in the patient-wise analysis and 67.26% and 99.60%, respectively, in the slice-wise analysis (Figs. 1 and 2).

### Reader assessment study

In the reader assessment study, the AI-assisted group exhibited a significantly higher diagnostic accuracy in detecting AIH than the AI-unassisted group for both patient-wise (0.9703 vs. 0.9471, *p* < 0.0001) and slice-wise analyses (0.9581 vs. 0.9522, *p* < 0.0001). Compared with the AI-unassisted group, the AI-assisted group achieved significantly higher sensitivity (0.9718 vs. 0.9437, *p* = 0.0003 for patient-wise analysis and 0.8469 vs. 0.8299, *p* < 0.0001 for slice-wise analysis) and specificity (0.9689 vs. 0.9504, *p* = 0.0145 for patient-wise analysis and 0.9855 vs. 0.9824, *p* < 0.0001 for slice-wise analysis) (Tables 2 and 3, Figs. 1 and 2).

**Table 2:** Accuracy, sensitivity, and specificity of each subgroup after conducting AI-assisted or AI-unassisted evaluation in patient-wise analysis.

Reviewers	AI-assisted (*N* = 296)	AI-unassisted (*N* = 296)	Difference (AI-assisted–AI-unassisted)	*p* value^a^
*Accuracy (%) (95% confidence interval)*				
Total reviewers	97.03 (96.32, 97.65)	94.71 (93.79, 95.53)	2.33 (1.26, 3.39)	<0.0001
Non-radiology physicians^b^	95.05 (93.40, 96.38)	91.89 (89.90, 93.60)	3.15 (0.86, 5.45)	0.0072
Board-certificated radiologists^c^	97.41 (96.14, 98.35)	94.59 (92.90, 95.99)	2.82 (1.00, 4.63)	0.0025
Neuroradiologists^d^	98.65 (97.65, 99.30)	97,64 (96.41, 98.53)	1.01 (−0.24, 2.27)	0.1138
*Sensitivity (%) (95% confidence interval)*				
Total reviewers	97.18 (96.29, 98.08)	94.37 (93.12, 95.61)	2.82 (1.28, 4.35)	0.0003
Non-radiology physicians^b^	96.12 (93.86, 97.72)	92.69 (89.84, 94.95)	3.42 (0.39, 6.46)	0.0274
Board-certificated radiologists^c^	96.58 (94.41, 98.07)	92.69 (89.84, 94.95)	3.88 (0.91, 6.85)	0.0108
Neuroradiologists^d^	98.86 (97.36, 99.63)	97.72 (95.84, 98.90)	1.14 (−0.57, 2.86)	0.1929
*Specificity (%) (95% confidence interval)*				
Total reviewers	96.89 (95.82, 97.75)	95.04 (93.74, 96.13)	1.85 (0.37, 3.34)	0.0145
Non-radiology physicians^b^	94.00 (91.39, 96.01)	91.11 (88.09, 93.57)	2.89 (−0.54, 6.31)	0.0988
Board-certificated radiologists^c^	98.22 (96.53, 99.23)	96.44 (94.29, 97.95)	1.78 (−0.32, 3.88)	0.0979
Neuroradiologists^d^	98.44 (96.82, 99.37)	97.56 (95.67, 98.77)	0.89 (−0.94, 2.72)	0.3409

*AI* artificial intelligence, *N* patient number.

^a^Chi-square test.

^b^Non-radiology physicians: 5–7 years of experience as a non-radiology physician.

^c^Board-certified radiologists: 5–7 years of experience as a radiologist.

^d^Neuroradiologists: 7–11 years of experience as a radiologist, including 3–7 years of experience as neuroradiologists.

**Table 3:** Accuracy, sensitivity, and specificity of each subgroup after conducting AI-assisted or AI-unassisted evaluation in slice-wise analysis.

Reviewers	AI-assisted (*N* = 12,663)	AI-unassisted (*N* = 12,663)	Difference (AI-assisted–AI-unassisted)	*p* value^a^
*Accuracy (%) (95% confidence interval)*				
Total reviewers	95.81 (95.69, 95.92)	95.22 (95.09, 95.34)	0.59 (0.42, 0.76)	<0.0001
Non-radiology physicians^b^	93.93 (93.69, 94.17)	93.06 (92.80, 93.32)	0.87 (0.52, 1.22)	<0.0001
Board-certificated radiologists^c^	96.42 (96.23, 96.61)	95.67 (95.46, 95.87)	0.75 (0.48, 1.03)	<0.0001
Neuroradiologists^d^	97.06 (96.89, 97.23)	96.91 (96.74, 97.09)	0.15 (−0.10, 0.39)	0.2345
*Sensitivity (%) (95% confidence interval)*				
Total reviewers	84.69 (84.21, 85.16)	82.99 (82.50, 83.48)	1.70 (1.02, 2.38)	<0.0001
Non-radiology physicians^b^	76.33 (75.35, 77.29)	76.00 (75.02, 76.96)	0.33 (−1.03, 1.69)	0.6324
Board-certificated radiologists^c^	88.40 (87.65, 89.11)	84.78 (83.95, 85.59)	3.62 (2.53, 4.70)	<0.0001
Neuroradiologists^d^	89.34 (88.62, 90.03)	88.20 (87.45, 88.92)	1.14 (0.13, 2.15)	0.0264
*Specificity (%) (95% confidence interval)*				
Total reviewers	98.55 (98.47, 98.63)	98.24 (98.15, 98.32)	0.32 (0.20, 0.43)	<0.0001
Non-radiology physicians^b^	98.28 (98.13, 98.42)	97.28 (97.09, 97.46)	1.00 (0.77, 1.24)	<0.0001
Board-certificated radiologists^c^	98.40 (98.26, 98.54)	98.36 (98.21, 98.50)	0.05 (−0.15, 0.25)	0.6531
Neuroradiologists^d^	98.97 (98.85, 99.08)	99.07 (98.95, 99.17)	−0.10 (−0.26, 0.06)	0.2176

*AI* artificial intelligence, *N* patient number.

^a^Chi-square test.

^b^Non-radiology physicians: 5–7 years of experience as a non-radiology physician.

^c^Board-certified radiologists: 5–7 years of experience as a radiologist.

^d^Neuroradiologists: 7–11 years of experience as a radiologist, including 3–7 years of experience as neuroradiologists.

Among the three subgroups of reviewers, the non-radiologist physicians demonstrated the greatest improvement in diagnostic accuracy with the use of AI assistance compared with that without AI assistance (0.9505 vs. 0.9189, with a difference of 3.15%, *p* = 0.0072 for patient-wise analysis and 0.9393 vs. 0.9306, with a difference of 0.87%, *p* < 0.0001 for slice-wise analysis). For the board-certified radiologists, AIH detection with AI assistance resulted in a significantly higher diagnostic accuracy compared with that without AI assistance (0.9741 vs. 0.9459, with a difference of 2.82%, *p* = 0.0025 for patient-wise analysis and 0.9632 vs. 0.9567, with a difference of 0.75%, *p* < 0.0001 for slice-wise analysis). For neuroradiologists, although AIH detection with AI assistance exhibited a trend for higher diagnostic accuracy compared with that without AI assistance, this did not reach statistical significance (0.9865 vs. 0.9764, with a difference of 1.01%, *p* = 0.1138 for patient-wise analysis and 0.9706 vs. 0.9691, with a difference of 0.15%, *p* = 0.2345 for slice-wise analysis) (Tables 2 and 3, Figs. 1 and 2). The diagnostic performance of the reviewers with basic ROC curves for AI standalone performance based on patient- and slice-wise analyses are presented in Figs. 1 and 2, respectively.

GEE analysis revealed that AI assistance resulted in a significant increase in accuracy in both patient- (3.67 for the AI-assisted group and 3.01 for the AI-unassisted group, with a difference of 0.66, *p* = 0.0075) and slice-wise analyses (3.21 for the AI-assisted group and 3.03 for the AI-unassisted group, with a difference of 0.18, *p* < 0.0001). Sensitivity increased significantly in both patient- (4.24 for the AI-assisted group and 2.89 for the AI-unassisted group, with a difference of 1.35, *p* = 0.017) and slice-wise analyses (1.75 for the AI-assisted group and 1.69 for the AI-unassisted group, with a difference of 0.05, *p* = 0.3273). Specificity also increased significantly in both patient- (3.81 for the AI-assisted group and 3.17 for the AI-unassisted group, with a difference of 0.364, *p* = 0.0376) and slice-wise analyses (4.56 for the AI-assisted group and 4.15 for the AI-unassisted group, with a difference of 0.41, *p* < 0.0001) (Supplementary Tables 4–7).

The ICC indicated that the AI-assisted and AI-unassisted groups demonstrated excellent (0.9193) and good (0.8475) reliability, respectively. Representative images of AIH detection from brain CT images are presented in Fig. 3 and Supplementary Fig. 1.

## Discussion

In the present study, we reported a new AI algorithm that uses a combination of supervised training for haemorrhage detection and unsupervised training for anomaly detection. In addition, we applied a joint CNN-RNN architecture for haemorrhage detection. Our AI algorithm achieved high accuracy for standalone AI detection, and its use in AI-assisted interpretation resulted in superior diagnostic performance in detecting AIH relative to interpretation without AI assistance.

With respect to the AUROC values, the performance of the standalone AI algorithm in the external validation study (0.992 and 0.977 in patient- and slice-wise analyses, respectively) and reader assessment study (0.9874 and 0.9671 in patient- and slice-wise analyses, respectively) were comparable with the performance of the neuroradiologist subgroup without AI assistance (0.9764 and 0.9691 in patient- and slice-wise analyses, respectively). These diagnostic accuracies were higher than those reported by the majority of previous studies^7,8,10,11,13,15^ and were comparable with the results achieved in a previous study (AUROC = 0.991), which reported that AI standalone performance was comparable with that of highly trained experts^13^. Furthermore, in the present study, the high sensitivity of 95.89% and specificity of 95.33% achieved by our approach at a cut-off level of 39.84% in the patient-wise analysis was higher than those achieved by reviewers without AI assistance (94.37% and 95.04%, respectively). The promising results achieved by our AI algorithm highlight its potential for the accurate detection of AIH on brain CT images.

In the reader assessment study, which employed a retrospective, multi-reader, pivotal, crossover, randomised study design, the AI-assisted group demonstrated a significantly higher diagnostic accuracy in detecting AIH than the AI-unassisted group. In addition, the superior performance of the AI-assisted group in terms of diagnostic accuracy was validated using GEE analysis. To the best of our knowledge, the beneficial effects of AI assistance in reader interpretation for the detection of AIH on brain CT images have not been previously reported. The promising findings in this study support the practical relevance of using AI in clinical settings to improve patient care. Notably, with the aid of our AI algorithm, the diagnostic performance of non-radiologist physicians reached the level for radiologists and the diagnostic performance of radiologists reached the level for neuroradiologists for the detection of AIH on brain CT images. We believe that our AI algorithm may play a key role as a reliable assistant in real-world clinical practice where prompt aid by expert radiologists or neuroradiologists may be unavailable. In addition, our AI algorithm may partly relieve the burden of radiologists and neuroradiologists who encounter large volumes of CT images that require interpretation with high diagnostic accuracy and efficiency in a timely manner. The significant improvement in sensitivity observed in this study implies that the present AI algorithm may reduce the occurrence of false negatives in which AIH may be erroneously excluded, thereby enabling prompt management that is critical for patients with AIH.

It is interesting to note that the difference between AI-assisted and AI-unassisted sensitivities shows a lower value for the slice-wise manner (1.70%) than that for the patient-wise manner (2.82%), and the improvement in terms of sensitivity for non-radiologist physicians in the patient-wise manner failed to achieve the statistical significance in the slice-wise manner (Tables 2 and 3). In addition, according to the GEE analysis, achievement of statistically significant superiority failed only in the analysis of sensitivity in slice-wise manner (Supplementary Table 6). The low sensitivity of AI standalone in the slice-wise manner (89.87%) compared with that in the patient-wise manner (95.89%) might make a consistent positive effect on the decision a challenge. In addition, the decrease in terms of the positive role might affect the non-radiology physicians group at a greater intensity. However, the statistically significant improvement of sensitivity in the neuroradiologists group only in the slice-wise manner remains a challenge that needs to be explained.

Although specificity was significantly improved in the AI-assisted group for all readers, we did not observe a statistically significant improvement in specificity for each group. This suggests that the ability of the present AI algorithm to reduce false positives may be limited and that our AI algorithm is more suitable as a supportive tool rather than an alternative method for the detection of AIH on brain CT images.

In the present study, we describe the development of a new AI algorithm, which combines haemorrhage detection and anomaly detection processes, with the aim of improving diagnostic performance for the identification of AIH on brain CT images. The majority of previous AI algorithms used to analyse medical imaging, including those designed for intracranial haemorrhage detection, have been developed using supervised labelling of training images to facilitate the biomarker detection process^7,8,10,11,13,15–17^. Although training using expert-labelled images has produced promising results^27,30,31^, discordance in labelled areas between experts is unavoidable. In addition, poorly defined characteristics, variation in sizes and morphologies, and the attenuation of AIH contribute to inter-observer discordance that may occur even between expert neuroradiologists. Anomaly detection is the process of identifying abnormal areas based on unsupervised training using normal data^21,22,32^. The application of anomaly detection based on unsupervised training using normal brain CT images may overcome the drawbacks of conventional AI algorithms for AIH detection that rely on supervised training. In the present study, the combination of haemorrhage detection and anomaly detection based on a relatively large dataset may have contributed to the superior performance demonstrated by the current AI algorithm.

To overcome the aforementioned issues and improve diagnostic performance, we used a combined CNN-RNN in our AI algorithm. With regard to deep learning architectures, previous studies have predominantly used algorithms based on 2D or 3D CNNs^11,13,23–26^. However, brain CT images consist of a series of 2D images that contain information about actual 3D structures. Therefore, in the present study, we designed an architecture that was more appropriate for processing 3D data and additionally applied an RNN module to the more common CNN module. The additional use of this RNN facilitated more accurate patient-wise AIH probability scores and improved diagnostic performance at both patient- and slice-wise levels.

Further work is warranted to address the utility of this AI algorithm from a clinical perspective, including investigations on related morbidity or mortality. In the present study, we addressed the diagnostic accuracy of the present AI algorithm in the detection of AIH on brain CT images; however, the critical characteristics of AIH evolution that are associated with clinical outcomes, including haemorrhage volume and expansion, require assessment with follow-up imaging to gain a full understanding of the diagnostic accuracy of our approach. As such, further investigations regarding the clinical utility of the present AI algorithm in patients with critical AIH for which clinical outcomes are available will clarify its potential role in diagnosing and managing this condition. In addition, the reading environment in this experimental study did not replicate that of daily practice, especially with regard to the use of clinical information. In clinical settings, patient information, including the chief complaints, symptoms, physical examination results, and past medical history contributed to superior diagnostic performance of the physicians. Therefore, the direct application of the present AI algorithm based on its excellent diagnostic performance in this experimental study may be premature. In addition, the classification of AIH by the gold-standard review board in this study may be a limitation. Determining the gold standard for AIH is challenging, particularly when the amount of haemorrhage is subtle such that no management is indicated, and further diagnostic steps, such as a lumbar puncture, would not be routinely considered, and may even be inaccurate. The ground truth may not be knowable in such cases in routine clinical medicine. To minimise the natural drawback in the diagnosis of AIH, in the present study, the gold standard for AIH classification was based on the interpretation of the gold-standard review board comprising three neuroradiologists with at least 11 years of relevant experience as radiologists, including at least 7 years of experience as neuroradiologists. However, achieving complete agreement between the two primary neuroradiologists was challenging. In the present study, the weighted kappa value for the inter-rater agreement between the experienced neuroradiologists was 0.9865, and two cases that were initially included in the AIH group were reclassified to the normal (without AIH) group. Although our approach to achieve a gold standard diagnosis was reasonable, there may be limitations in terms of the appropriateness of our method for identifying the gold standard used for validation of the AI algorithm, which achieved a diagnostic accuracy of up to 0.9874 according to these decisions. Finally, demographic traits of the included cases and retrospective design of the study that allows for possible selection bias are additional limitations.

In conclusion, we developed a deep learning-based AI algorithm for automatic AIH detection on brain CT images based on a combination of a haemorrhage detection process, which employed a combined CNN-RNN architecture, and an anomaly detection process, which used unsupervised training. The diagnostic performance of the AI algorithm was validated in a large external validation dataset. Additionally, the improvement in diagnostic performance with AI assistance versus that without AI assistance was also validated in this retrospective multi-reader study.

## Methods

### Study design

We developed and validated a deep learning-based AI algorithm (Medical Insight+ Brain Hemorrhage, SK Inc. C&C, Seongnam, Republic of Korea) for automatic AIH detection on brain CT scans. This study was approved by the institutional review boards of the participating institutions (H-2007-061-1140, Seoul National University Hospital Institutional Review Board [institution A] and AJIRB-DEV-DE3-20-379, Ajou University Medical Center Institutional Review Board [institution B]), and the requirement for informed consent was waived owing to the retrospective nature of this study.

### Development dataset

To develop the AI algorithm for use with our diagnostic support software, 104,666 slices (28,351 [27.1%] with AIH and 76,315 [72.9%] without AIH) from 3010 patients (2010 [66.8%] with AIH and 1000 [33.2%] without AIH) from two institutions (Seoul National University Hospital [institution A] and Ajou University Medical Center [institution B]) were used for model development. Data were collected from patients in institutions A and B between April 2009 and December 2015 and between April 2004 and April 2020, respectively. AIH at the underlying pathology (including intratumoural haemorrhage and haemorrhagic transformation at the site of acute ischaemic stroke) as well as solitary AIH were also enrolled in the AIH group. Most of the development dataset (2632 among total 3010 patients [87 4%]) had a slice thickness of 5 mm (2 5 mm [*n* = 3], 3.0 mm [*n* = 104], 3.75 mm [*n* = 1], 4.0 mm [*n* = 40], 4.5 mm [*n* = 209], 4.8 mm [*n* = 12], 5.3125 mm [*n* = 1], 6.0 mm [*n* = 4], and 7.0 mm [*n* = 4]).

### External validation dataset

For the external validation of the diagnostic performance of the AI algorithm, 1,855,465 slices (73,467 [4 0%] with AIH and 1,781,998 [96.0%] without AIH) from 49,841 patients (6442 [12.9%] with AIH and 43,399 [87.1%] without AIH) in the AI hub under the direction of the Korean National Information Society Agency (https://aihub.or.kr/aidata/34101) were used. This dataset was collected from six medical institutions in Korea in 2020 as a big data collection project on cerebrovascular disease, and the hospitals contributing to the data collection for the AI hub are different from the hospitals from which the development dataset was collected. The decision regarding whether all 1,855,465 slices from 49,841 patients were either AIH or normal was made based on the image interpretation by the neuroradiologists at each institution. A total of 6442 CT images showed AIH, including 2424 cases of subarachnoid haemorrhage, 2738 cases of subdural haemorrhage, 371 cases of epidural haemorrhage, 1266 cases of intraventricular haemorrhage, and 3367 cases of intraparenchymal haemorrhage (note: overlapping subtypes were possible). A total of 73,467 slices exhibited AIH, including 32,751 cases of subarachnoid haemorrhage, 39,604 cases of subdural haemorrhage, 4567 cases of epidural haemorrhage, 18,220 cases of intraventricular haemorrhage, and 35,669 cases of intraparenchymal haemorrhage (note: overlapping subtypes were possible). A summary of the patient and scanner information regarding the external validation is presented in Supplementary Tables 8 and 9.

### Reader study dataset

A dataset temporally separated from the development dataset was obtained for reader assessment. A total of 12,663 brain (2508 AIH [19 8%] and 10,155 normal [81.2%]) from 296 patients (146 AIH [49 3%] and 150 normal [51.7%]) CT slices were obtained from two institutions (Seoul National University Hospital [institution A] and Ajou University Medical Center [institution B]). Data were collected from patients in institutions A and B between January 2016 and December 2019 and between April 2004 and April 2020, respectively. Patients enrolled in the development dataset were not enrolled in the reader study dataset.

All 296 complete CT images that satisfied the criteria for image quality modified from previously reported criteria were enrolled as the dataset for the reader assessment study (Supplementary Table 10)^33,34^. The number of required CT images was calculated using the power estimation method with the significance level set to 5% and the power to 90%, which was based on a sensitivity of 88.6% as reported previously^27^ and a sensitivity of 98.5% from internal validation of the present AI algorithm. This resulted in a total of 148 CT images for each group while accounting for a 15% dropout rate. In addition, based on a specificity of 88.6% reported in a previous study^27^ and a specificity of 96.0% from internal validation of the present AI algorithm, 114 CT images for each group were obtained while accounting for a 15% dropout rate.

The gold standard for interpretation of all 12,663 slices from 296 CT images as either AIH or normal was achieved via careful consensus of a gold-standard review board comprising three neuroradiologists with at least 11 and 7 years of experience as radiologists and neuroradiologists, respectively. For CT interpretation, two radiologists independently interpreted the presence or absence of AIH in both a patient-wise and slice-wise manner. A third neuroradiologist reviewed the cases for which there was a disagreement between the two initial neuroradiologists to make a final decision. The weighted kappa value of the inter-rater agreement between the initial independent interpretations by the experienced neuroradiologists was 0.9865 [95% CI: 0.9732, 0.9997] for patient-wise analysis and was based on the interpretations of the gold-standard review board. Two cases that had initially been categorised in the AIH group according to medical records were reclassified to the normal group. In total, 146 CT images exhibited AIH, including 101 cases of subarachnoid haemorrhage, 72 cases of subdural haemorrhage, 20 cases of epidural haemorrhage, 40 cases of intraventricular haemorrhage, and 66 cases of intraparenchymal haemorrhage (note: overlapping subtypes were possible). A total of 2508 slices exhibited AIH, including 1408 cases of subarachnoid haemorrhage, 1150 cases of subdural haemorrhage, 228 cases of epidural haemorrhage, 240 cases of intraventricular haemorrhage, and 535 cases of intraparenchymal haemorrhage (note: overlapping subtypes were possible). A summary of the reader study population is presented in Supplementary Table 11.

### Development of the AI algorithm

For AI algorithm development, 28,351 slices from 2010 patients with AIH and 1000 normal participants were annotated by neuroradiologists using nordicICE version 4.1.3 (NordicNeuroLab, Bergen, Norway), with a particular focus on AIH areas. To overcome the drawbacks of inter-observer variability by supervised training, we developed a new AI algorithm based on a combination of a supervised haemorrhage detection process and an unsupervised anomaly detection process.

The purpose of the haemorrhage detection process is to predict whether AIH is present on brain CT images. This process consists of two modules^15,27,35^. The first is a CNN-based haemorrhage detection module that provides the feature vector and AIH score for the target. The second is an RNN-based sequence module with double layers. In this module, more accurate AIH scores for each slice are produced using the feature vectors and scores from the first module as inputs to overcome the limitations of CNNs in terms of 3D image data analysis. In addition, scores for each patient were acquired simultaneously.

An anomaly detection process was applied to predict whether anomalies were present on brain CT images. A generation module based on a variational auto-encoder^36,37^ and a generative adversarial network^38^ was used in this process. The generation module was trained to generate normal CT slices (restored CT images) using images from the normal group. As such, a comparison of restored and input CT images indicated areas of anomaly when considering areas presumed to have AIH in the haemorrhage detection process.

Finally, AI-assisted brain CT images, which included an embedded heatmap depicting the probable location of AIH according to patient- and slice-wise AIH probability scores, were provided to the picture archiving and communication system (PACS) viewer alongside original brain CT images (Fig. 1). An overview and details of the AI algorithm architecture are presented in Fig. 4 and Supplementary Figs. 2 and 3.

### Diagnostic performance of the AI-based diagnostic support software in the external validation dataset

Per-patient and per-slice AIH probability scores were used to evaluate the standalone performance metrics of our AI algorithm, including the accuracy, sensitivity, specificity, positive predictive value, negative predictive value, F1 score, and area under the receiver operating characteristic curve (AUROC).

### Evaluation of the diagnostic performance of the AI-based diagnostic support software

Per-patient and per-slice AIH probability scores were used to evaluate the standalone performance metrics of our AI algorithm, including the AUROC, sensitivity, and specificity.

### Reader assessment study

A retrospective, multi-reader, crossover, superiority, pivotal, randomised study was performed to evaluate the efficacy of the software assisting the diagnosis decision with respect to the identification and detection of intracranial haemorrhage on brain CT scans (Clinical Research Information Service of Republic of Korea [https://cris.nih.go.kr; identifier: KCT0006734], which is a Korean primary registry of the World Health Organization’s International Clinical Trials Registry Platform that is under the direction of the Korea Disease Control and Prevention Agency) (Supplementary Note (Study Details)).

This retrospective multi-reader study was conducted with nine reviewers from four institutions in South Korea (Seoul National University Hospital, Ajou University Medical Center, Bundang Seoul National University Hospital, and Seongnam Medical Center) using 12,663 brain CT slices from 296 patients as the study dataset. Nine physicians from three different subgroups with equal numbers (i.e., three non-radiologist physicians with 5–7 years of experience in that role, three board-certified radiologists with 5–7 years of experience in that role, and three subspecialty-trained neuroradiologists with 7–11 years of experience as radiologists, including 3–7 years of experience as neuroradiologists) participated as reviewers.

**Fig. 5:**
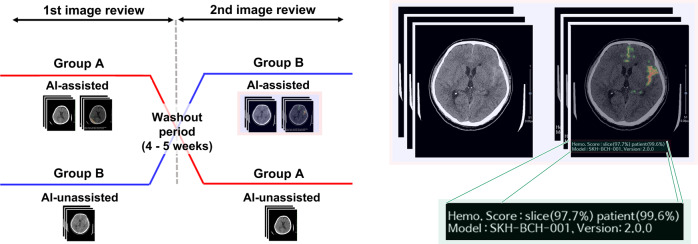
Schematic overview of study design. The schematic diagram shows the retrospective, pivotal, crossover, randomised study design used in the present study (left). In the first image review, group A consisted of original CT images and corresponding AI-assisted CT images, while group B consisted of only the original CT images without AI-assisted CT images. After a washout period of 4–5 weeks, in the second image review, the group A dataset was changed to include only the original CT images without any AI-assisted CT images, while AI-assisted CT images were added to the group B dataset. The AI-assisted CT images provided a heatmap with information on the suspected location and probability of AIH in a patient- and slice-wise manner (right).

In this retrospective, multi-reader, pivotal, crossover, randomised study, prior to the first assessment, the full CT dataset was split into groups A and B, each comprising CT images from 148 patients, and numbers for sequential assessment were randomly assigned. Group A consisted of original CT images and corresponding AI-assisted CT images, while group B consisted of only original CT images without AI-assisted CT images. The AI-assisted CT images provided a heatmap with information on the suspected location of AIH and probability of AIH in a patient- and slice-wise manner. Each reviewer independently reviewed the CT images for the detection of AIH. The PACS image viewer was used to assess CT images in a patient- and slice-wise manner. The reviewers were blinded to the decisions of the gold-standard review board with regard to AIH and proportion of AIH cases in the assessed dataset. After a washout period of 4–5 weeks, a second assessment was conducted. In the second assessment, the group A dataset comprising original and AI-assisted CT images during the first assessment was changed to include only the original CT images without any AI-assisted CT images, whereas AI-assisted CT images were added to the group B dataset that had previously included only the original CT images without AI-assisted CT images. The numbers for sequential assessments were randomly re-assigned. Each reviewer repeated the same review process as per the first assessment. A schematic overview of the study design is presented in Fig. 5.

### Statistical analysis

AI determination was based on whether the probability provided by the AI algorithm was equal or over the cut-off level. For external validation, a decision was considered correct if the AI determination matched the suggested decision made on the basis of the basic information on the external validation dataset; sensitivity and specificity were calculated at a cutoff level of 50.0%. However, for standalone AI assessment, a decision was considered correct if the AI determination matched the decision made by the gold-standard review board for AUROC analysis; sensitivity and specificity were also calculated at a cutoff level of 50.0%.

In the reader study, the correctness of a decision was determined based on whether the decision of the reader matched the decision made by the gold-standard review board. Sensitivity, specificity, and accuracy were compared between AI-assisted and AI-unassisted groups using the chi-square test. To validate the superior performance of the AI-assisted group as compared with that of the AI-unassisted group, logistic regression using the generalised estimating equation (GEE) method was used for significance testing and for estimating the 95% confidence intervals (CIs). Inter-observer agreement according to AIH subtype was analysed using an intra-class correlation coefficient based on a patient-wise analysis. All analyses were performed using SAS statistical software (version 9.4; SAS Institute, Cary, NC, USA).

### Reporting summary

Further information on research design is available in the Nature Research Reporting Summary linked to this article.

## Supplementary information

Supplementary Information

REPORTING SUMMARY

## Data Availability

Additional documents related to this study are available from the corresponding author upon reasonable request. The datasets from Seoul National University Hospital and Ajou University Medical Center were used under license for the current study and are not publicly available.

## Code availability

The code used to train the AI model is dependent on annotation, infrastructure, and hardware; thus, it cannot be released. However, all experimental and implementation details that can be shared are described in detail in the Supplementary Note (Study Details). The AI algorithm developed from this study is available through the commercial product, SK Inc. C&C Medical Insight+ Brain Hemorrhage.
