# Supplementary material for: Deep learning based automatic detection algorithm for acute intracranial haemorrhage: a pivotal randomized clinical trial
Source: NPJ Digit Med. 2023 Apr 7;6:61. doi: 10.1038/s41746-023-00798-8 (PMC10082037; doi:10.1038/s41746-023-00798-8)
Supplement: Supplementary file 1 — Supplementary Information [file 41746_2023_798_MOESM1_ESM.pdf]

# Supplementary Information

**Supplementary Table 1. Sensitivity of AI in the external validation set according to subtypes of AIH (AIH = acute intracranial haemorrhage, SAH = subarachnoid haemorrhage, SDH = subdural haemorrhage EDH = epidural haemorrhage, IVH = intraventricular haemorrhage, IPH = intraaparenchymal haemorrhage, PPV = positive predictive value, AUC = area under the reciever-operating curve, NPV = negative predictive value)**

| | | **Subtypes of AIH*** | | | | |
|---|---|---|---|---|---|---|
| | | **SAH** | **SDH** | **EDH** | **IVH** | **IPH** |
| Patient-wise (N = 6,442) | No. | 2,424 | 2,738 | 371 | 1,266 | 3,367 |
| | Sensitivity | 0.954 | 0.933 | 0.933 | 0.994 | 0.977 |
| Slice-wise (N = 73,467) | No. | 32,751 | 39,604 | 4,567 | 18,220 | 35,669 |
| | Sensitivity | 0.821 | 0.731 | 0.775 | 0.874 | 0.854 |

* Overlapping subtypes are possible.

**Supplementary Table 2. Diagnostic performance of AI in the external validation set according to equipment (PPV = positive predictive value, AUC = area under the reciever-operating curve, NPV = negative predictive value)**

| Model | | No. | **Accuracy** | **Recall (Sensitivity)** | **Precision (PPV)** | **F1 Score** | **Specificity** | **AUC** | **NPV** |
|---|---|---|---|---|---|---|---|---|---|
| GE DISCOVERY CT750 HD | Patient | 5,999 | 0.983 | 0.946 | 0.925 | 0.935 | 0.989 | 0.988 | 0.992 |
| | Slice | 190,682 | 0.988 | 0.846 | 0.864 | 0.855 | 0.994 | 0.991 | 0.993 |
| GE LIGHTSPEED VCT | Patient | 123 | 0.983 | 0.946 | 0.925 | 0.935 | 0.989 | 0.996 | 0.978 |
| | Slice | 4,645 | 0.983 | 0.810 | 0.755 | 0.782 | 0.989 | 0.992 | 0.992 |
| GE Optima CT660 | Patient | 9,194 | 0.972 | 0.951 | 0.879 | 0.914 | 0.976 | 0.993 | 0.991 |
| | Slice | 319,570 | 0.989 | 0.855 | 0.891 | 0.872 | 0.995 | 0.996 | 0.993 |
| GE Revolution | Patient | 31 | 0.968 | 1.000 | 0.800 | 0.889 | 0.963 | 1.000 | 1.0 |
| | Slice | 2,310 | 0.986 | 0.905 | 0.576 | 0.704 | 0.988 | 0.993 | 0.998 |
| GE Others | Patient | 33 | 0.939 | | 0 | | 0.939 | | 1 |
| | Slice | 2,686 | 0.998 | | 0 | | 0.998 | | 1 |
| Philips BRILLIANCE 16 | Patient | 2 | 1.000 | 1.000 | 1.000 | 1.000 | 1.000 | 1.000 | 1.0 |
| | Slice | 80 | 0.986 | 0.905 | 0.576 | 0.704 | 0.988 | 0.993 | 0.83 |
| Philips BRILLIANCE 64 | Patient | 448 | 0.980 | 1.000 | 0.471 | 0.640 | 0.980 | 0.997 | 1.0 |
| | Slice | 14,402 | 0.996 | 0.653 | 0.636 | 0.645 | 0.998 | 0.998 | 0.998 |
| Philips BRILLIANCE 64 | Patient | 12 | 1.0 | | | | 1.0 | | 1.0 |
| | Slice | 700 | 1.0 | | | | 1.0 | | 1.0 |
| Philips ICT 256 | Patient | 8,469 | 0.977 | 0.933 | 0.696 | 0.798 | 0.979 | 0.989 | 0.996 |
| | Slice | 270,630 | 0.993 | 0.808 | 0.810 | 0.809 | 0.997 | 0.994 | 0.996 |
| Philips INGENUITY | Patient | 1,162 | 0.994 | 1.000 | 0.125 | 0.222 | 0.994 | 0.999 | 1.0 |
| | Slice | 38,765 | 1.000 | 0.667 | 0.133 | 0.222 | 1.000 | 1.0 | 1.0 |
| | Patient | 217 | 0.954 | 0.938 | 0.625 | 0.750 | 0.955 | 0.981 | 0.995 |

| Model | | No. | Accuracy | Recall (Sensitivity) | Precision (PPV) | F1 Score | Specificity | AUC | NPV |
|---|---|---|---|---|---|---|---|---|---|
| Philips IQON-SPECTRAL | Slice | 7,037 | 0.987 | 0.739 | 0.756 | 0.747 | 0.994 | 0.994 | 0.993 |
| Philips Others | Patient | 5 | 1.0 | | | | 1.0 | | 1.0 |
| | Slice | 266 | 1.0 | | | | 1.0 | | 1.0 |
| Siemens SENSATION 64 | Patient | 5,732 | 0.973 | 0.949 | 0.986 | 0.967 | 0.990 | 0.995 | 0.963 |
| | Slice | 191,277 | 0.961 | 0.845 | 0.894 | 0.869 | 0.982 | 0.985 | 0.972 |
| Siemens SOMATOM DEFINITION | Patient | 691 | 0.941 | 0.946 | 0.909 | 0.927 | 0.937 | 0.988 | 0.963 |
| | Slice | 29,647 | 0.921 | 0.616 | 0.674 | 0.643 | 0.961 | 0.874 | 0.95 |
| Siemens SOMATOM DEFINITION AS+ | Patient | 590 | 0.966 | 0.939 | 0.912 | 0.925 | 0.974 | 0.991 | 0.982 |
| | Slice | 23,857 | 0.947 | 0.575 | 0.641 | 0.606 | 0.975 | 0.901 | 0.968 |
| Siemens SOMATOM DEFINITION EDGE | Patient | 13,180 | 0.982 | 0.897 | 0.694 | 0.782 | 0.985 | 0.987 | 0.996 |
| | Slice | 587,243 | 0.992 | 0.589 | 0.628 | 0.608 | 0.996 | 0.927 | 0.995 |
| Siemens SOMATOM DEFINITION FLASH | Patient | 1,081 | 0.969 | 0.903 | 0.873 | 0.888 | 0.980 | 0.985 | 0.985 |
| | Slice | 47,891 | 0.983 | 0.693 | 0.869 | 0.771 | 0.996 | 0.98 | 0.987 |
| Siemens SOMATOM DRIVE | Patient | 14 | 1.0 | | | | 1.0 | | 1.0 |
| | Slice | 568 | 1.0 | | | | 1.0 | | 1.0 |
| Siemens SOMATOM FORCE | Patient | 682 | 0.955 | 0.981 | 0.886 | 0.931 | 0.942 | 0.995 | 0.991 |
| | Slice | 27,840 | 0.946 | 0.679 | 0.720 | 0.699 | 0.973 | 0.912 | 0.968 |
| Siemens SOMATOM PERSPECTIVE | Patient | 12 | 1.0 | | | | 1.0 | | 1.0 |
| | Slice | 700 | 1.0 | | | | 1.0 | | 1.0 |
| Siemens Others | Patient | 1,889 | 0.987 | 0.952 | 0.738 | 0.831 | 0.989 | 0.995 | 0.998 |
| | Slice | 80,354 | 0.988 | 0.433 | 0.495 | 0.462 | 0.995 | 0.871 | 0.993 |
| Toshiba AQUILION ONE | Patient | 1 | 1.0 | | | | 1.0 | | 1.0 |
| | Slice | 32 | 1.0 | | | | 1.0 | | 1.0 |
| Toshiba Others | Patient | 249 | 0.972 | 1.000 | 0.500 | 0.667 | 0.971 | 0.993 | 1.0 |
| | Slice | 12,271 | 0.995 | 0.860 | 0.454 | 0.594 | 0.995 | 0.995 | 0.999 |
| Others | Patient | 37 | 0.919 | 0.0 | 0.0 | 0.0 | 0.944 | 0.722 | 0.971 |
| | Slice | 2,712 | 0.999 | 0.0 | 0.0 | 0.0 | 1.000 | 0.933 | 0.999 |

**Supplementary Table 3. Diagnostic performance of AI in the external validation set according to slice thickness (PPV = positive predictive value, AUC = area under the reciever-operating curve, NPV = negative predictive value)**

| Slice thickness | | No. | Accuracy | Recall (Sensitivity) | Precision (PPV) | F1 Score | Specificity | AUC | NPV |
|---|---|---|---|---|---|---|---|---|---|
| ≤ 4 mm | Patient | 18,539 | 0.978 | 0.927 | 0.820 | 0.870 | 0.983 | 0.991 | 0.994 |
| | Slice | 831,530 | 0.985 | 0.619 | 0.677 | 0.646 | 0.993 | 0.927 | 0.991 |
| > 4 mm | Patient | 31,302 | 0.976 | 0.949 | 0.904 | 0.926 | 0.981 | 0.992 | 0.99 |
| | Slice | 1,023,935 | 0.986 | 0.850 | 0.884 | 0.866 | 0.994 | 0.994 | 0.992 |

**Supplementary Table 4. Clinical sensitivity between AI-unassisted and AI-assisted evaluations based on patient-wise analysis (full assessment set: 296 patients) (GEE = generalised estimating equation)**

| GEE Result (sensitivity) | AIH (N = 146) | | |
|---|---|---|---|
| | AI-assisted (N = 146) | AI-unassisted (N = 146) | p-value* |
| n/N | 141.89/146 | 137.78/146 | |
| Estimate | 4.24 | 2.89 | 0.0017 |
| 95% Confidence Interval | (3.44, 5.04) | (2.43, 3.35) | |
| 95% Confidence Interval for difference (AI-assisted – AI-unassisted) | 1.35 (0.51, 2.19) | | |

* Chi-square test

**Supplementary Table 5. Clinical specificity between AI-unassisted and AI-assisted evaluations based on patient-wise analysis (full assessment set: 296 patients) (GEE = generalised estimating equation)**

| GEE Result (specificity) | AIH (N = 150) | | |
|---|---|---|---|
| | AI-assisted (N = 150) | AI-unassisted (N = 150) | p-value* |
| n/N | 145.33/150 | 142.56/150 | |
| Estimate | 3.81 | 3.17 | 0.0376 |
| 95% Confidence Interval | (3.24, 4.38) | (2.73, 3.61) | |
| 95% Confidence Interval for difference (AI-assisted – AI-unassisted) | 0.64 (0.04, 1.24) | | |

* Chi-square test

**Supplementary Table 6. Clinical sensitivity between AI-unassisted and AI-assisted evaluations based on slice-wise analysis (full assessment set: 12,663 slices) (GEE = generalised estimating equation)**

| GEE Result (sensitivity) | AIH (N = 2,508) | | |
|---|---|---|---|
| | AI-assisted (N = 2,508) | AI-unassisted (N = 2,508) | p-value* |
| n/N | 2,124/2,508 | 2,081.44/2,508 | |
| Estimate | 1.75 | 1.69 | 0.3273 |
| 95% Confidence Interval | (1.67, 1.83) | (1.62, 1.77) | |
| 95% Confidence Interval for difference (AI-assisted – AI-unassisted) | 0.05 (-0.05, 0.16) | | |

* Chi-square test

**Supplementary Table 7. Clinical specificity between AI-unassisted and AI-assisted evaluations based on slice-wise analysis (full assessment set: 12,663 slices) (GEE = generalised estimating equation)**

| GEE Result (specificity) | Normal (N = 2,508) | | |
|---|---|---|---|
| | AI-assisted (N = 2,508) | AI-assisted (N = 2,508) | p-value* |
| n/N | 10,007.89/10,155 | 9,975.78/10,155 | |
| Estimate | 4.56 | 4.15 | <0.0001 |
| 95% Confidence Interval | (4.44, 4.67) | (4.06, 4.23) | |
| 95% Confidence Interval for difference (AI-assisted – AI-unassisted) | 0.41 (0.29, 0.53) | | |

* Chi-square test

**Supplementary Table 8. Patient information and characteristics of CT data selected for the external validation set (full analysis set: 49,841 patients) (AIH = acute intracranial haemorrhage)**

| | AIH (N = 6,442) | Non-AIH (N = 43,399) | Total (N = 49,841) |
|---|---|---|---|
| **Sex, N (%)** | | | |
| Men | 3,775 (14.84) | 21,658 (85.16) | 25,433 (51.03) |
| Women | 2,667 (10.93) | 21,741 (89.07) | 24,408 (48.97) |
| **Age (years)** | | | |
| Mean (SD) | 61.6 (17.9) | 53.5 (21.9) | 54.5 (21.6) |
| Median | 64.00 | 57.00 | 58.00 |
| Min, Max | 2.00, 98.00 | 2.00, 100.00 | 2.00, 100.00 |

**Supplementary Table 9. Scanner information and characteristics of CT data selected for the external validation set (full analysis set: 49,841 patients)**

| Equipment | Number of patient | | | | | |
|---|---|---|---|---|---|---|
| | H1 (n = 14,344) | H2 (n = 2,932) | H3 (n = 6,595) | H4 (n = 9,347) | H5 (n = 10,512) | H6 (n = 6,121) |
| GE MEDICAL SYSTEMS DISCOVERY CT750 HD | 231 | | | | | 5,768 |
| GE MEDICAL SYSTEMS LIGHTSPEED VCT | 26 | | | 97 | | |
| GE MEDICAL SYSTEMS OPTIMA CT660 | 20 | | | 7,739 | 1435 | |
| GE MEDICAL SYSTEMS REVOLUTION | 29 | | | | 2 | |
| GE MEDICAL SYSTEMS OTHERS | 33 | | | | | |
| PHILIPS BRILLIANCE 16 | 2 | | | | | |

| Equipment | Number of patient | | | | | |
|---|---|---|---|---|---|---|
| | H1<br>(n = 14,344) | H2<br>(n = 2,932) | H3<br>(n = 6,595) | H4<br>(n = 9,347) | H5<br>(n = 10,512) | H6<br>(n = 6,121) |
| PHILIPS BRILLIANCE 64 | 13 | | | 21 | 414 | |
| PHILIPS ICT 256 | 25 | | | | 8,444 | |
| PHILIPS INGENUITY | 52 | | | 1,110 | | |
| PHILIPS IQON - SPECTRAL CT | | | | | 217 | |
| PHILIPS OTHERS | 5 | | | | | |
| SIEMENS SENSATION 64 | 196 | | 5,536 | | | |
| SIEMENS SOMATOM DEFINITION | 563 | | | 128 | | |
| SIEMENS SOMATOM DEFINITION AS+ | 441 | | | | | 149 |
| SIEMENS SOMATOM DEFINITION EDGE | 10,334 | 2,846 | | | | |
| SIEMENS SOMATOM DEFINITION FLASH | 31 | | 1,050 | | | |
| SIEMENS SOMATOM DRIVE | 3 | | | | | 11 |
| SIEMENS SOMATOM FORCE | 339 | 86 | 8 | 249 | | |
| SIEMENS SOMATOM PERSPECTIVE | 6 | | | | | 6 |
| SIEMENS OTHERS | 1,888 | | 1 | | | |
| TOSHIBA AQUILION ONE | 1 | | | | | |
| TOSHIBA OTHERS | 63 | | | | | 186 |
| OTHERS | 33 | | | 3 | | 1 |

**Supplementary Table 10. Criteria for image quality in full assessment set (full analysis set: 296 patients)**

| 5-point scale | Criteria | N (%) |
|---|---|---|
| 1 | Non diagnostic due to excessive noise or artifacts | 0 (0.00) |
| 2 | Diagnosis questionable due to excessive noise or artifacts, moderate decrease in confidence | 0 (0.00) |
| 3 | Diagnostic with moderate but acceptable noise or artifacts | 44 (14.86) |
| 4 | Mild noise, no change in confidence | 107 (36.15) |
| 5 | Routine diagnostic image quality | 145 (48.99) |

**Supplementary Table 11. Patient information and characteristics of CT data selected for the reader assessment study (full analysis set: 296 patients, 12,663 slices) (AIH = acute intracranial haemorrhage)**

| | AIH (N = 146) | Non-AIH (N = 150) | Total (N = 296) |
|---|---|---|---|
| **Sex, N (%)** | | | |
| Men | 97 (66.44) | 73 (48.67) | 170 (57.43) |
| Women | 49 (33.56) | 77 (51.33) | 126 (42.57) |
| **Age (years)** | | | |
| Mean (SD) | 56.92 (16.65) | 57.27 (18.38) | 57.10 (17.52) |
| Median | 58.00 | 57.00 | 58.00 |
| Min, Max | 21.00, 94.00 | 20.00, 97.00 | 20.00, 97.00 |
| **Slice Number** | | | |
| N | 6188 | 6475 | 12663 |
| Mean (SD) | 42.38 (10.89) | 43.17 (11.75) | 42.78 (11.32) |
| Median | 46.00 | 36.00 | 41.50 |
| Min, Max | 27.00, 62.00 | 27.00, 72.00 | 27.00, 72.00 |
| **Patient Type, N (%)** | | | |
| Emergency | 136 (93.15) | 99 (66.00) | 235 (79.39) |
| Inpatient | 10 (6.85) | 19 (12.67) | 29 (9.80) |
| Outpatient | 0 (0.00) | 32 (21.33) | 32 (10.81) |

## Supplementary Note (Study Details)

### 1. Background

| | |
|---|---|
| CRIS Registration Number | KCT0006734 |
| Unique Protocol ID | SKAIIHC-01 |
| Public/Brief Title | Multicentre, randomised, retrospective, crossover, superiority, pivotal study to evaluate the efficacy of 'SKH-BCH-001' software assisting the diagnosis decision regarding to identification and detection of intracranial haemorrhage on Brain CT scans |
| Scientific Title | Multicentre, randomised, retrospective, crossover, superiority, pivotal study to evaluate the efficacy of 'SKH-BCH-001' software assisting the diagnosis decision regarding to identification and detection of intracranial haemorrhage on Brain CT scans |
| Acronym | |
| MFDS Regulated Study | Yes |
| IND/IDE Protocol | Yes |

| | |
|---|---|
| Registered at Other Registry | No |
| Healthcare Benefit Approval Status | Submitted approval |

2. **Institutional Review Board / Ethics Committee**

| | |
|---|---|
| Board Approval Status | Submitted approval |
| Board Approval Number | AJIRB-DEV-DE3-20-379 |
| Approval Date | 2020-10-29 |
| Institutional Review Board Name | Ajou University Hospital Institutional Review Board |
| Institutional Review Board Address | 164, World cup-ro, Yeongtong-gu, Suwon-si, Gyeonggi-do |
| Institutional Review Board Telephone | +82-31-219-5569 |

3. **Contact Details**

- **Contact Person for Principal Investigator / Scientific Queries**

| | |
|---|---|
| Name | Jinwook Choi |
| Title | Professor |
| Telephone | +82-31-219-5852 |
| Affiliation | Ajou University Hospital |
| Address | (16499) 164, World cup-ro,Yeongtong-gu, Suwon, Korea |

- **Contact Person for Public Queries**

| | |
|---|---|
| Name | Okkyung Kang |
| Title | Manager |
| Telephone | +82-2-6400-0114 |
| Affiliation | SK |
| Address | 26,Jong-ro,Jongno-gu,Seoul,03188 |

- **Contact Person for Updating Information**

| | |
|---|---|
| Name | Okkyung Kang |
| Title | Manager |
| Telephone | +82-2-6400-0114 |
| Affiliation | SK |
| Address | 26,Jong-ro,Jongno-gu,Seoul,03188 |

4. **Status**

| | |
|---|---|
| Study Site | Multi-centre Number of centre : 2 |
| Overall Recruitment Status | Completed |
| Date of First Enrolment | 2020-12-02 Actual |
| Target Number of Participant | 296 |
| Primary Completion Date | 2021-02-18 , Actual |

- **Recruitment Status by Participating Study Site 1**

| | |
|---|---|
| Name of Study | Seoul National University Hospital |

| | |
|---|---|
| Recruitment Status | Completed |
| Date of First Enrolment | 2020-12-02 , |

- **Recruitment Status by Participating Study Site 2**

| | |
|---|---|
| Name of Study | Ajou University Hospital |
| Recruitment Status | Completed |
| Date of First Enrolment | 2020-12-02 , |

### 5. Source of Monetary / Material Support

| | |
|---|---|
| Organisation Name | SK |
| Organisation Type | Others |
| Project ID | SKAIICH-01 |

### 6. Sponsor Organisation

| | |
|---|---|
| Organisation Name | Ajou University Hospital |
| Organisation Type | Medical Institute |

### 7. Study Summary

| | |
|---|---|
| Lay Summary | 1. Purpose of Clinical Trial<br>This clinical trial was designed to evaluate the efficacy of 'SKH-BCH-001' a software that assists medical staff in determining the priority of brain haemorrhage diagnosis by analysing brain computed tomography (CT) images without contrast agent in slice units, and then using an artificial intelligence algorithm based on a convolutional neural network (CNN) to segment brain regions and model abnormal regions, and by detecting the location with a high probability of brain haemorrhage.<br>The primary purpose of this clinical trial is to prove that the clinical sensitivity and specificity of the medical equipment(software)<br>2. Background<br>Intracranial haemorrhage can be diagnosed by using computed tomography (CT) and magnetic resonance imaging (MRI). CT scan is useful because it has the advantage of being able to proceed with the test relatively quickly and quickly discriminating whether or not there is a intracranial haemorrhage. However, certain factors of CT, such as signal to noise, signal attenuation, and artifacts, may have a negative effect on diagnosing a lesion and thus may lead to misdiagnosis. According to previous studies, discrepancies between the initial and final diagnosis results may lead to misdiagnosis. The discrepancy result for intracranial haemorrhage accounted for 13.6% of these results, and among them, the misdiagnosed type of intracranial haemorrhage consisted of 39.0% of traumatic subdural haemorrhage (SDH) and 33.0% of subarachnoid haemorrhage (SAH), respectively. Therefore, it is necessary to reduce the misdiagnosis of intracranial haemorrhage and increase the diagnostic efficacy. |

| | |
|---|---|
| | Accordingly, SK Inc. has developed 'SKH-BCH-001', an automated prioritisation software that assists medical staff in determining the priority of brain haemorrhage diagnosis by analysing brain computed tomography (CT) images without contrast agent in slice units, and then using an artificial intelligence algorithm based on a convolutional neural network (CNN) to segment brain regions and model abnormal regions, and by detecting the location with a high probability of brain haemorrhage. It is expected that the efficacy of diagnosis will increase as it can make a quick diagnosis.<br>3.Medical devices for clinical trials<br>- Testing equipment for clinical trials Medical Insight + Brain Haemorrhage (SKH-BCH-001)<br>Purpose of Use: The product is a software that assists medical staff in determining the priority of brain haemorrhage diagnosis by analysing brain computed tomography (CT) images without contrast agent in slice units, and then using an artificial intelligence algorithm based on a convolutional neural network (CNN) to segment brain regions and model abnormal regions, and by detecting the location with a high probability of brain haemorrhage.<br>4. Clinical trial period: it is expected that it will take a total of 3 months, including 1 month for medical data collection and screening and 2 months for image reading. It is also expected that it will take an additional 4 months to prepare the report for results.<br>5. Target images: Brain CT image taken for diagnosis of intracranial haemorrhage (ICH).<br>6. Number of target images: A total of 296 images (148 images of intracranial haemorrhage and 148 images of normal patients).<br>7. Clinical trial design: Multicentre, randomized, retrospective, confirmatory<br>8. Clinical trial method<br>Observing the accuracy when using AI and not using AI for 9 image readers to verify the accuracy improvement effect when using AI<br>9. Efficacy Assessment Variables<br>- AUC of ROC Curve (Area Under the Curve of Receiver Operating Characteristic Curve) for the algorithm of the test device.<br>- Sensitivity for algorithm of the test device (Clinical Sensitivity, %)<br>- Specificity for algorithm of the test device (Clinical Specificity, %) |

## 8. Study Design

| | |
|---|---|
| Study Type | Interventional Study |
| Study Purpose | Diagnosis |
| Phase | Phase2 |
| Intervention Model | Cross-over |
| Blinding/Masking | Single |
| Blinded Subject | Investigator |
| Allocation | RCT |
| Intervention Type | Medical Device |

<table>
<tr><td colspan="2">Intervention Description</td><td>9 image readers* will read all the images allocated to Group A or Group B as follows, and record the reading results in the source document. When the image reading is completed, the source document is delivered to the data manager. At this time, the source document should be composed in such a file format that only the data administrator can access.<br>*9 image readers consist of 3 neuro-radiologists with 7-11 years of experience, 3 board certified radiologists with 5-7 years of experience, and 3 non-radiology physicians with 5-7 years of experience.<br>- At this time, the data are blinded so that the image reader cannot know the reference standard diagnosis result.<br>Image Reading Procedure<br>In the case of Group A, images are read under the conditions of use of medical devices for clinical trials (test group). After the washout period (2 weeks or more), images are read under the condition of not using the clinical trial medical device (control group). Images in Group B are read in the reverse order.<br>- Before the second image reading, the investigator reassigns the image to a randomised number allocated to the second reading and delivers it to 9 image readers</td></tr>
<tr><td colspan="2">Number of Arms</td><td>2</td></tr>
<tr><td rowspan="4">Arm 1</td><td>Arm Label</td><td>Test Group</td></tr>
<tr><td>Target Number of Participant</td><td>148</td></tr>
<tr><td>Arm Type</td><td>Experimental</td></tr>
<tr><td>Arm Description</td><td>The image is read under the conditions of using medical devices for clinical trials</td></tr>
<tr><td rowspan="4">Arm 2</td><td>Arm Label</td><td>Control Group</td></tr>
<tr><td>Target Number of Participant</td><td>148</td></tr>
<tr><td>Arm Type</td><td>No intervention</td></tr>
<tr><td>Arm Description</td><td>The image is read under the condition that the medical device for clinical trial is not used.</td></tr>
</table>

## 9. Study Eligibility

<table>
<tr><td colspan="2">Condition(s)/Problem(s)</td><td>* (I00-I99)Diseases of the circulatory system<br>(I60.8)Other subarachnoid haemorrhage<br><br>Intracranial haemorrhage Bleeding within the SKULL, including haemorrhages in the brain and the three membranes of MENINGES. The escape of blood often leads to the formation of HEMATOMA in the cranial epidural, subdural, and subarachnoid spaces.</td></tr>
<tr><td colspan="2">Rare Disease</td><td>No</td></tr>
<tr><td rowspan="2">Inclusion Criteria</td><td>Gender</td><td>Both</td></tr>
<tr><td>Age</td><td>19Year~99Year</td></tr>
</table>

| | | |
|---|---|---|
| | Description | All the following selection criteria must be satisfied to participate in this clinical trial.<br>1. Brain CT images of men and women over 19 years of age.<br>2. Brain CT image taken for examination of suspected intracranial haemorrhage (ICH).<br>3. Images that can secure clinical information* of the image provider.<br>* Clinical information: Image acquisition year, number of slices, gender, date of birth, patient type (emergency, inpatient, outpatient)<br>- Except for personally identifiable information.<br>4. Images with a score of 3 or higher in the image quality survey (refer to the image quality survey criteria table)<br>Image quality survey criteria table<br>5-point scale Criteria<br>1 Non diagnostic due to excessive noise or artifacts<br>2 Diagnosis questionable due to excessive noise or artifacts, moderate decrease in confidence<br>3 Diagnostic with moderate but acceptable noise or artifacts<br>4 Mild noise, no change in confidence<br>5 Routine diagnostic image quality<br>Ref) Fletcher et al. AJNR Am. J. Neruoradiol (2019), Fletcher et al. Radiology (2015)<br><br>* 0.5 mm ≤ z-axial slice thickness (mm) ≤ 5 mm |
| Exclusion Criteria | | If any of the following exclusion criteria are met, images cannot be used for this clinical trial.<br>1. Brain CT imaging using a contrast medium<br>2. Brain CT image that has already been used as a training set or validation set for the development of medical devices for clinical trials.<br>- In order to collect the images for this clinical trial as independent data apart from the training set or validation set, Seoul National University Hospital will use brain CT images excluding images taken from January 2009 to December 2015 as of the date of image recording, and Ajou University hospitals will use images taken on all dates, but for images from April 2004 to April 2020 as of the date of image recording, only the data, whose independence is confirmed by checking the image list provided to the institution for image identification, will be used as images for this clinical trial.<br>3. In case the principal investigator determines that participation in the clinical trial is inappropriate because it affects the efficacy assessment result or for other reasons (the specific reason is recorded in the case record). |
| Healthy Volunteers | | No |

**Supplementary Figure 1. Overview of AI algorithm – haemorrhage detection**

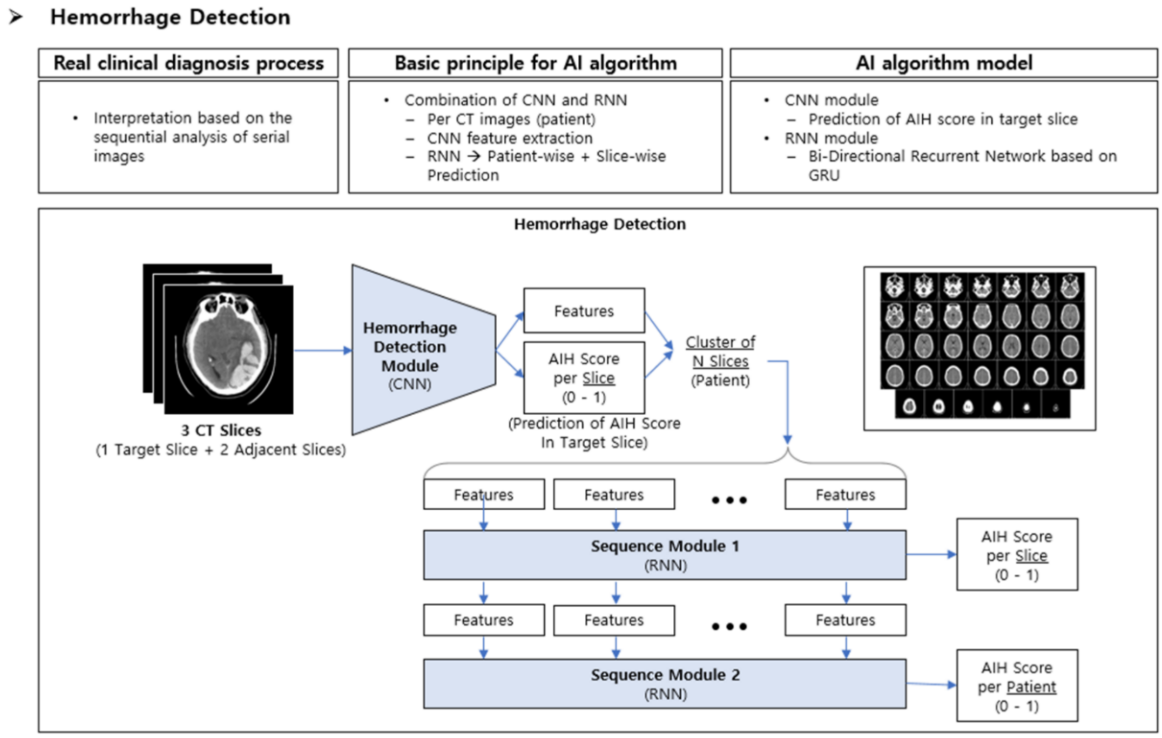


**Supplementary Figure 2. Overview of AI algorithm – anomaly detection**

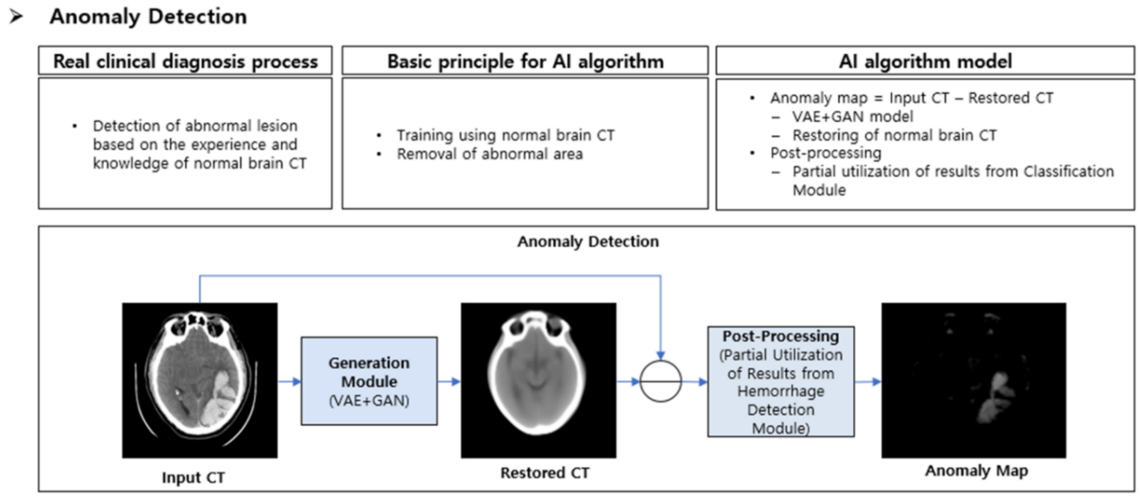

**Supplementary Figure 3. Representative images**

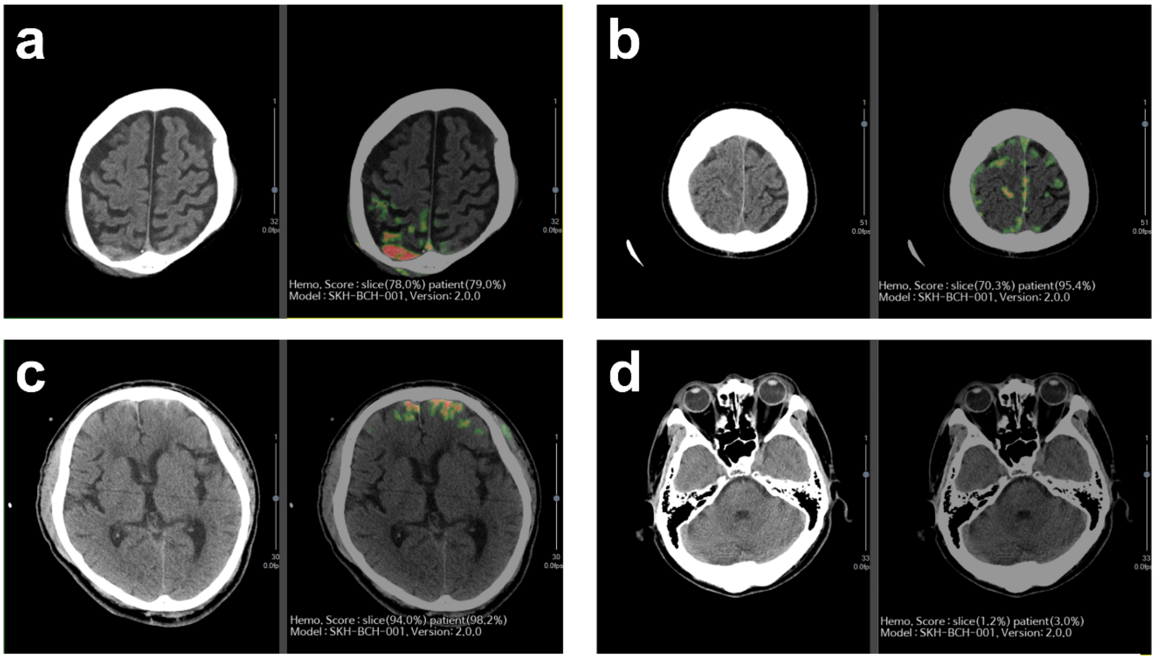


a. AI-assisted brain CT shows the likely location of epidural haemorrhage in the right parietal convexity and provides a slice-wise AIH probability score (78.0%) and a patient-wise AIH probability score (79.0%). After interpretation without AI assistance, two non-radiologist physicians missed this case of AIH. However, with AI assistance, these reviewers were able to correctly revise their decision.

b. AI-assisted brain CT shows the likely location of subarachnoid haemorrhage in the right vertex area and provides a slice-wise AIH probability score (70.3%) and a patient-wise AIH probability score (95.4%). After interpretation without AI assistance, two non-radiologist physicians and one board-certified radiologist missed this case of AIH. However, with AI assistance, these reviewers were able to correctly revise their decision.

c. AI-assisted brain CT shows the likely location of subarachnoid haemorrhage in the bilateral frontal sulci and provides a slice-wise AIH probability score (94.0%) and a patient-wise AIH probability score (98.2%). After interpretation without AI assistance, one board-certified radiologist missed this case of AIH. However, with AI assistance, this reviewer was able to correctly revise their decision.

d. The AI-assisted brain CT shows that the curvilinear hyperdense lesion in the right posterior fossa is probably a normal finding (such as right transverse sinus) rather than a case of AIH and provides a low

slice-wise AIH probability score (1.2%) and a low patient-wise AIH probability score (3.0%). After interpretation without AI assistance, one non-radiologist physician reported this as a subdural haemorrhage. However, with AI assistance, this reviewer was able to correctly revise their decision.
