# Supplementary material for: Deep learning based automatic detection algorithm for acute intracranial haemorrhage: a pivotal randomized clinical trial
Source: NPJ Digit Med. 2023 Apr 7;6:61. doi: 10.1038/s41746-023-00798-8 (PMC10082037; doi:10.1038/s41746-023-00798-8)
Supplement: Supplementary file 2 — REPORTING SUMMARY [file 41746_2023_798_MOESM2_ESM.pdf]

nature portfolio

Corresponding author(s): Jin Wook Choi/NPJDIGITALMED-04897R

Last updated by author(s): Feb 25, 2023

Reporting Summary

Nature Portfolio wishes to improve the reproducibility of the work that we publish. This form provides structure for consistency and transparency in reporting. For further information on Nature Portfolio policies, see our Editorial Policies and the Editorial Policy Checklist.

Statistics

For all statistical analyses, confirm that the following items are present in the figure legend, table legend, main text, or Methods section.

n/a	Confirmed	
☐	☒	The exact sample size (n) for each experimental group/condition, given as a discrete number and unit of measurement
☐	☒	A statement on whether measurements were taken from distinct samples or whether the same sample was measured repeatedly
☐	☒	The statistical test(s) used AND whether they are one- or two-sided<br>*Only common tests should be described solely by name; describe more complex techniques in the Methods section.*
☒	☐	A description of all covariates tested
☐	☒	A description of any assumptions or corrections, such as tests of normality and adjustment for multiple comparisons
☐	☒	A full description of the statistical parameters including central tendency (e.g. means) or other basic estimates (e.g. regression coefficient) AND variation (e.g. standard deviation) or associated estimates of uncertainty (e.g. confidence intervals)
☐	☒	For null hypothesis testing, the test statistic (e.g. F, t, r) with confidence intervals, effect sizes, degrees of freedom and P value noted<br>*Give P values as exact values whenever suitable.*
☐	☒	For Bayesian analysis, information on the choice of priors and Markov chain Monte Carlo settings
☒	☐	For hierarchical and complex designs, identification of the appropriate level for tests and full reporting of outcomes
☐	☒	Estimates of effect sizes (e.g. Cohen's d, Pearson's r), indicating how they were calculated

Our web collection on statistics for biologists contains articles on many of the points above.

Software and code

Policy information about availability of computer code

Data collection	No software was used.
Data analysis	The AI algorithm developed from this study is available through the commercial product, SK Inc. C&C Medical Insight+ Brain Hemorrhage.All analyses were performed using SAS statistical software (version 9.4; SAS Institute, Cary, NC, USA).

For manuscripts utilizing custom algorithms or software that are central to the research but not yet described in published literature, software must be made available to editors and reviewers. We strongly encourage code deposition in a community repository (e.g. GitHub). See the Nature Portfolio guidelines for submitting code & software for further information.

Data

Policy information about availability of data

All manuscripts must include a data availability statement. This statement should provide the following information, where applicable:

- Accession codes, unique identifiers, or web links for publicly available datasets
- A description of any restrictions on data availability
- For clinical datasets or third party data, please ensure that the statement adheres to our policy

Additional documents related to this study are available from the corresponding author upon reasonable request. The datasets from Seoul National University Hospital and Ajou University Medical Center were used under license for the current study and are not publicly available.

Human research participants

Policy information about studies involving human research participants and Sex and Gender in Research.

Reporting on sex and gender	This study is a retrospective analysis study using patient CT images. Gender considerations are based on the biological sex of patients enrolled in the hospital.
Population characteristics	For the external validation set, AIH (N=6442, M:F=3775:2667, Age=mean,61.6;SD,17.9) and Non-AIH (N=43399, M:F=21658:21741, Age=mean,53.5;SD,21.9). For the reader assessment study, AIH (N=146, M:F=97:49, Age=mean,56.92;SD,16.65) and Non-AIH (N=150, M:F=73:77, Age=mean,57.27;SD,18.38).
Recruitment	Data were collected from patients in institutions A and B between January 2016 and December 2019 and between April 2004 and April 2020, respectively.
Ethics oversight	This study was approved by the institutional review boards of the participating institutions (AJIRB-DEV-DE3-20-379, Ajou University Hospital Institutional Review Board), and the requirement for informed consent was waived owing to the retrospective nature of this study.

Note that full information on the approval of the study protocol must also be provided in the manuscript.

Field-specific reporting

Please select the one below that is the best fit for your research. If you are not sure, read the appropriate sections before making your selection.

☒ Life sciences ☐ Behavioural & social sciences ☐ Ecological, evolutionary & environmental sciences

For a reference copy of the document with all sections, see nature.com/documents/nr-reporting-summary-flat.pdf

Life sciences study design

All studies must disclose on these points even when the disclosure is negative.

Sample size	All 296 complete CT images that satisfied the criteria for image quality modified from previously reported criteria were enrolled as the dataset for the reader assessment study. The number of required CT images was calculated using the power estimation method with the significance level set to 5% and the power to 90%, which was based on a sensitivity of 88·6% as reported previously and a sensitivity of 98·5% from internal validation of the present AI algorithm. This resulted in a total of 148 CT images for each group while accounting for a 15% dropout rate. In addition, based on a specificity of 88·6% reported in a previous study and a specificity of 96·0% from internal validation of the present AI algorithm, 114 CT images for each group were obtained while accounting for a 15% dropout rate.
Data exclusions	1. Brain CT imaging using a contrast medium<br>2. Brain CT image that has already been used as a training set or validation set for the development of medical devices for clinical trials.
Replication	In this retrospective, multi-reader, pivotal, crossover, randomised study, prior to the first assessment, the full CT dataset was split into groups A and B, each comprising CT images from 148 patients, and numbers for sequential assessment were randomly assigned. Group A consisted of original CT images and corresponding AI-assisted CT images, while group B consisted of only original CT images without AI-assisted CT images. The AI-assisted CT images provided a heatmap with information on the suspected location of AIH and probability of AIH in a patient- and slice-wise manner. Each reviewer independently reviewed the CT images for the detection of AIH. The PACS image viewer was used to assess CT images in a patient- and slice-wise manner. The reviewers were blinded to the decisions of the gold-standard review board with regard to AIH and proportion of AIH cases in the assessed dataset. After a washout period of 4–5 weeks, a second assessment was conducted. In the second assessment, the group A dataset comprising original and AI-assisted CT images during the first assessment was changed to include only the original CT images without any AI-assisted CT images, whereas AI-assisted CT images were added to the group B dataset that had previously included only the original CT images without AI-assisted CT images. The numbers for sequential assessments were randomly re-assigned. Each reviewer repeated the same review process as per the first assessment.
Randomization	In the case of Group A, images are read under the conditions of use of medical devices for clinical trials (test group). After the washout period (2 weeks or more), images are read under the condition of not using the clinical trial medical device (control group). Images in Group B are read in the reverse order.<br>- Before the second image reading, the investigator reassigns the image to a randomised number allocated to the second reading and delivers it to 9 image readers
Blinding	All images were evaluated in anonymized state, and all evaluators evaluated in a separate space.

Reporting for specific materials, systems and methods

We require information from authors about some types of materials, experimental systems and methods used in many studies. Here, indicate whether each material, system or method listed is relevant to your study. If you are not sure if a list item applies to your research, read the appropriate section before selecting a response.

Materials & experimental systems

n/a	Involved in the study
☒	☐ Antibodies
☒	☐ Eukaryotic cell lines
☒	☐ Palaeontology and archaeology
☒	☐ Animals and other organisms
☐	☒ Clinical data
☒	☐ Dual use research of concern

Methods

n/a	Involved in the study
☒	☐ ChIP-seq
☒	☐ Flow cytometry
☒	☐ MRI-based neuroimaging

Clinical data

Policy information about clinical studies

All manuscripts should comply with the ICMJE guidelines for publication of clinical research and a completed CONSORT checklist must be included with all submissions.

Clinical trial registration	Clinical Research Information Service of Republic of Korea [https://cris.nih.go.kr; identifier: KCT0006734]
Study protocol	Clinical Research Information Service of Republic of Korea [https://cris.nih.go.kr; identifier: KCT0006734]
Data collection	Data were collected from patients in institutions A and B between January 2016 and December 2019 and between April 2004 and April 2020, respectively.
Outcomes	- AUC of ROC Curve (Area Under the Curve of Receiver Operating Characteristic Curve) for the algorithm of the test device.<br>- Sensitivity for algorithm of the test device (Clinical Sensitivity, %)<br>- Specificity for algorithm of the test device (Clinical Specificity, %)
